# Skeletal muscle fatigue resistance is differentially affected in 8‐month‐old male and female hAβ‐KI mice

**DOI:** 10.14814/phy2.71153

**Published:** 2026-10-20

**Authors:** Emily N. Copeland, Evelin Melekh, Val Andrew Fajardo, Rebecca E. K. MacPherson

**Affiliations:** ^1^ Department of Kinesiology Brock University St. Catharines Ontario Canada; ^2^ Department of Health Sciences Brock University St. Catharines Ontario Canada; ^3^ Centre for Bone and Muscle Health Brock University St. Catharines Ontario Canada; ^4^ Centre for Neurosciences Brock University St. Catharines Ontario Canada

**Keywords:** age, amyloid beta, mouse, sex, skeletal muscle

## Abstract

Approximately 95% of Alzheimer's disease (AD) diagnoses are sporadic onset, present after the age of 65, and arising from a combination of non‐modifiable and modifiable factors. This underscores the potential to identify early systemic changes that may contribute to disease development. One factor may be skeletal muscle atrophy, which has been associated with cognitive decline, although the exact relationship between skeletal muscle and the brain is unknown. Furthermore, whether muscle atrophy begins prior to or after the onset of AD remains elusive. The humanized amyloid‐beta knock‐in mouse (hAβ‐KI) exhibits age‐related cognitive decline, insoluble Aβ accumulation, inflammation and microglial activation, making it a useful model of sporadic AD. However, characterization of skeletal muscle changes with aging has yet to be examined. Here, we assess skeletal muscle form and function of the hAβ‐KI mouse at 8 and 15 months of age, before and after onset of cognitive decline, through assessment of whole‐body lean mass percentage, isolated muscle contraction of the extensor digitorum longus (EDL) and soleus muscle, and fiber type analysis of male and female mice. Whole‐body lean mass declined over time in all mice; however, only male hAβ‐KI mice had a significant change in body mass and lean mass over the study. At 8‐months‐old, the isolated EDL of male hAβ‐KI mice fatigued faster than the WT mice, but there were no differences at 15 months‐old nor fiber type composition at either age. Alternatively, the isolated EDL and soleus of 8‐month‐old hAβ‐KI female mice fatigued slower compared to the WT mice, and there were no differences at 15 months nor fiber type composition at either age. This preliminary characterization work suggests sex‐dependent differences in isolated muscle fatigue prior to cognitive decline in the hAβ‐KI mouse model of sporadic AD.

## 1 INTRODUCTION

Alzheimer's disease (AD) is the most common form of dementia, accounting for approximately 60%–80% of all diagnoses. AD is characterized by progressive neuronal degeneration, cognitive impairments, and the gradual but significant deposition of its pathological hallmarks amyloid beta (Aβ) plaques and neurofibrillary tangles. AD has two patterns of onset: familial AD, which accounts for less than 5% of all AD diagnoses and manifests before the age of 65 (Zhu et al., 2015), and sporadic AD, which accounts for the remaining 95% of diagnoses and develops after the age of 65. While familial AD is heavily linked to autosomal dominant mutations (Price & Sisodia, 1998; Tanzi et al., 1996), sporadic AD is associated with both non‐modifiable and modifiable risk factors including, but not limited to, age, sex, diet, and physical inactivity (Moradi et al., 2020; Mosconi et al., 2018; Yau et al., 2025).

Emerging evidence suggests that skeletal muscle may be a modulator of brain health and given the prolonged preclinical phase of AD, skeletal muscle health may play a vital role in AD pathogenesis. The age‐related muscle loss (sarcopenia) begins as early as the third decade of life, progressing at a rate of decline of approximately 3%–5% per decade and accelerating after the age of 60 (Volpi et al., 2004). This timeline overlaps substantially with the preclinical phase of AD, which can span 10–20 years prior to clinical diagnosis and often begins midlife (~40 years of age) (Dubois et al., 2016; Jack et al., 2013). Further, sarcopenia has been linked to several comorbidities including cardiovascular disease and type 2 diabetes, and AD (Beeri et al., 2021; Mesinovic et al., 2019; Zuo et al., 2023). Specific to AD, previous work has highlighted that the prevalence of sarcopenia is significantly higher in AD patients compared to non‐AD controls (Ogawa et al., 2018). Furthermore, older adults diagnosed with sarcopenia are more likely to develop cognitive impairments, including poorer performance on tests of executive function, calculations, and recall, with performance declining as sarcopenia worsens (Dong et al., 2024).

Preclinical models of AD have shown similar relationships between AD pathology and skeletal muscle wasting. Hindlimb casting, leading to skeletal muscle atrophy, of young, presymptomatic 5×FAD mice worsened performance on memory tests compared to non‐casted 5×FAD mice, suggesting accelerated cognitive decline due to muscle wasting (Nagase & Tohda, 2021). Meanwhile, 18–20‐month‐old triple transgenic AD female mice exhibit lower skeletal muscle mass and poorer neuromuscular junction function and composition compared to their wildtype (WT) counterparts (Xu et al., 2022). However, much of the current research regarding the relationship between skeletal muscle and brain health in preclinical AD models comes from models of fAD (Brisendine et al., 2024; Monteiro‐Cardoso et al., 2015), and thus is only representative of approximately 5% of the population diagnosed with AD, bringing about a need for a more translatable model.

Recently, a mouse model with a humanized amyloid beta (Aβ) peptide‐coding sequence in the mouse amyloid precursor protein (App) gene was developed to generate a non‐mutant human Aβ knock‐in (hAβ‐KI) (Baglietto‐Vargas et al., 2021). The hAβ‐KI mouse exhibits age‐dependent changes to memory and increased Aβ production beginning around 10–14 months of age (Baglietto‐Vargas et al., 2021). As it has no familial genetic manipulation, the hAβ‐KI mouse is thought to be a novel model of sAD, and is therefore more translatable to the wider population of those diagnosed with AD. However, though the cognitive effects of the hAβ‐KI mouse have been well characterized upon its generation (Baglietto‐Vargas et al., 2021), the peripheral effects – particularly on skeletal muscle – are yet to be explored. Here, we aim to characterize skeletal muscle size and strength, endurance, and fiber type composition of both male and female hAβ‐KI mice. Furthermore, we conducted this characterization at 8 and 15 months of age to determine whether muscle function and composition change before or after the previously observed cognitive decline at 10 and 14 months, respectively (Baglietto‐Vargas et al., 2021).

## 2 METHODS

### 2.1 Animals

5‐week‐old male hAβ‐KI (APP +/+) mice were bred with 5‐week‐old female C57BL6/N7 mice to produce 100% heterozygous offspring. These heterozygous mice were then bred to produce WT (APP −/−), APP heterozygous (APP −/+), and APP homozygous (APP +/+) offspring. WT and APP +/+ (hAβ‐KI) offspring were used for further characterization. Mice were randomly separated into two age cohorts and aged to 8 months of age or 15 months of age (*n* = 20 per group and *n* = 10 per sex per age cohort). All mice were housed in Brock University's Animal Facility in an environmentally controlled room with a standard 12:12 h light–dark cycle. Mice were allowed access to food (Teklad Global 14% Protein Rodent Maintenance Diet, Envigo; 2014) and water ad libitum. Food was weighed once a week to assess intake. All experimental procedures were approved by Brock University's Animal Care Committee (AUP No. 23‐08‐02) and carried out in accordance with the Canadian Council on Animal Care guidelines.

### 2.2 DXA scans

Beginning at 4 months of age, lean and fat mass percentages were measured using a small animal InSIGHT dual X‐ray absorptiometry (DXA) scanner (Scintica Instrumentation Inc., London, ON) and were repeated every 2 months until the completion of the study, as previously described (Melekh et al., 2026). Briefly, two X‐ray images were taken, one on low energy and one on high energy, and measured at both energy levels to determine fat mass and non‐bone/lean mass. Mice were anesthetized (vaporized isoflurane) in a knockdown chamber prior to being placed on the scanner. Once on the scanner, mice were fitted with a nose cone attached to an isoflurane vaporizer and eye drops were administered to protect from dry eye.

### 2.3 Tissue collection

At 8 and 15 months of age, mice were euthanized via exsanguination under general anesthesia (vaporized isoflurane) and their tissues and serum collected. Left and right soleus and extensor digitorum longus (EDL) muscles were isolated, with one each being frozen and stored at −80°C for further analysis, and the other used for contractility and force production measures. Following contraction and force production protocols, muscles were embedded in optimal cutting temperature (OCT) compound, frozen, and stored at −80°C for subsequent histological and immunofluorescent fiber type analysis.

### 2.4 Isolated muscle contractility

Soleus and EDL muscles underwent a contractility protocol as previously described (Whitley et al., 2020) to assess isometric force production and fatigue. Briefly, muscles were mounted onto an Aurora Scientific contractile apparatus (model 305B and 701B). Muscles were then subjected to an isometric force frequency protocol consisting of 1, 20, 40, 80, and 160 Hz stimulation (450 ms, separated by 1 min) to obtain peak isometric force. Two minutes after the 160 Hz stimulation frequency, muscles underwent a fatigue protocol consisting of 70 Hz volleys (450 ms) every 2 s for 3 min. Peak isometric force was normalized to muscle cross sectional area (CSA), which is calculated using the formula: CSA = m/l*d*(L_f_/L_0_), where m is muscle mass in milligrams (mg), l is muscle length in millimeters (mm), d is the mammalian skeletal muscle density (1.06 mg/mm^3^; (Méndez & Keys, 1960)), and L_f_/L_0_ is the fiber length‐to‐muscle length ratio (0.71 for the soleus; 0.45 for the EDL; (Brooks & Faulkner, 1988)). For fatigue, data is presented as percent of the initial force, with the area‐under‐the‐curve (AUC) being analyzed, representing the total force generated over time.

### 2.5 Immunofluorescent fiber typing

Fiber typing was performed via immunofluorescent staining of myosin heavy chain isoforms in the soleus and EDL muscle as previously described (Bloemberg & Quadrilatero, 2012). Briefly, mounted OCT muscles were cut into 10 uM‐thick cryosections with a cryostat maintained at −20°C. Following slicing, slides were blinded prior to analysis. Sections were incubated for 1 h in blocking solution (10% goat serum in 1X phosphate‐buffered saline (PBS)). Primary antibodies were diluted in 10% goat serum (MHC I (BA‐F8) 1:50; MHC IIa (SC‐71) 1:600; MHC IIb (BF‐F3) 1:100) and incubated overnight. Slides were then washed 3× with 1X PBS and incubated with secondary antibody for 1 h in the dark (Alexa Fluor 350 IgG_2b_ (blue; type I fibers) 1:500; Alexa Fluor 488 IgG_1_ (green; type IIa fibers) 1:500; Alexa Fluor 555 IgM (red; type IIb) 1:1000). The slides were washed 3× with 1X PBS, the ProLong Gold Antifade Reagent was added, and a coverslip was mounted. Slides were imaged using a BioTek Cytation 5 Multimode Plate Reader at 10× magnification using auto‐exposure settings for three filters: DAPI, GFP, and Texas Red. Images were stitched, processed, and saved using Gen5 image processing functions and analyzed using ImageJ (NIH) software to estimate the fiber type composition and CSA. Fiber type percent composition was measured by counting the number of fibers of each fiber type (type I being blue, type IIa being green, type IIb being bright red, and type IIx being faint red) using the cell counter tool and dividing each count by the total number of fibers across a whole muscle. CSA was measured by averaging the areas of 25 fibers of each type. In the case where 25 fibers of a particular fiber type were not present, the maximum number of fibers was analyzed and averaged to obtain a CSA value.

### 2.6 RNA sequencing

The EDL from both 8‐ and 15‐month‐old male and female WT and hAβ‐KI mice (*n* = 3 per group) were homogenized in 1 mL of TRIzol Reagent (15596026, ThermoFisher Scientific, MI, USA). RNA was extracted using an Invitrogen PureLink RNA Mini Kit (12183205, ThermoFisher Scientific). Total RNA sequencing with purified mRNA was performed by Novogene (Novogene Corporation Inc., Sacramento, CA, USA) using NovaSeqXPlus. Differentially expressed genes (DEGs) between male WT versus hAβ‐KI and female WT versus hAβ‐KI at each age group were identified using the following thresholds: |log2FC| > 0.58 and *p* < 0.05, which correspond to 50% gene expression changes, as reported by Novogene using the DESeq2 software (Love et al., 2014). DEGs were then subjected to gene ontology (GO) pathway enrichment analysis, using NovoMagic, an online RNA‐Seq Bioinformatics Tool (Novogene), with select statistically significant pathways presented (based on adjusted *p* < 0.05 after Benjamini‐Hochberg correction). All up‐ and down‐regulated genes, as well as significant GO pathways, are listed in Datasets S1–, S4.

### 2.7 Statistical analysis

For body composition (DXA) and food intake, statistical analysis was performed using two‐way ANOVAs with main effects of genotype and age and genotype and sex, respectively. A two‐way repeated measures ANOVA was performed for all contractility analysis, examining main effects of frequency and genotype for absolute and specific force production, and main effects of time and genotype for fatigue. A Sidak's post hoc test was used for significant interactions. A Student's *t*‐test was used to compare all other analyses. For all data, 8‐ and 15‐month data was stratified and examined independently for each sex. Stratification by sex is a sex‐based statistical approach recommended by the Canadian Institutes of Health Research. All analysis was performed using GraphPad Prism 10 (San Diego, USA) software. Statistical outliers were identified using the ROUT method within GraphPad Prism, with *Q* = 5% across all experiments. Significance was set to *p* < 0.05. All values are represented as means ± SD.

## 3 RESULTS

### 3.1 Body composition

To assess changes in lean mass percentage over the course of the study, all mice were subjected to DXA scans every 2 months. There was a significant main effect of time in the male mice, in which both male WT and hAβ‐KI mice exhibited higher body mass, lower lean mass percentage, and higher fat mass percentage with age (Table 1). Similarly, both the WT and hAβ‐KI female mice showed a significant main effect of time, in which all mice exhibited higher body mass, lower lean mass percentage, and higher fat mass percentage with age (Table 2). We also investigated the change in body mass, lean mass, and fat mass between the genotypes. The male hAβ‐KI mice exhibited larger gains in body mass alongside a greater loss in lean mass percentage compared to WT mice between baseline and 15‐month measurements; however, there were no significant differences between the female mice (Table 3). The changes in body composition could not be explained by food intake as there were no significant differences across the study between WT and hAβ‐KI mice (Figure 1).

**TABLE 1:** Male body composition.

	4 months			15 months		
	Body mass (g)	Lean mass %	Fat mass %	Body mass (g)	Lean mass %	Fat mass %
WT	29.9 ± 2.45	76.4 ± 3.89	21.7 ± 3.49	39.1 ± 3.20^a^	73.5 ± 2.52^a^	25.0 ± 2.45^a^
hAβ‐KI	30.4 ± 4.06	78.4 ± 3.76	20.0 ± 3.73	42.1 ± 5.10^a^	72.7 ± 2.99^a^	25.8 ± 2.95^a^

^a^ *p* <0.0001 versus 4 months. Comparisons were made between time periods and genotypes using a two‐way ANOVA.

**TABLE 2:** Female body composition.

	4 months			15 months		
	Body mass (g)	Lean mass %	Fat mass %	Body mass (g)	Lean mass %	Fat mass %
WT	23.6 ± 1.14	82.0 ± 2.58	16.1 ± 2.48	34.9 ± 4.86^a^	73.4 ± 4.11^a^	24.9 ± 4.17^a^
hAβ‐KI	22.4 ± 1.60	81.8 ± 2.16	16.1 ± 1.49	34.6 ± 4.57^a^	73.5 ± 3.06^a^	24.8 ± 3.07^a^

^a^ *p* <0.0001 versus 4 months. Comparisons were made between time periods and genotypes using a two‐way ANOVA.

**TABLE 3:** Change in body composition between 4 and 15 months.

	Males			Females		
	Δ body mass (g)	Δ lean mass %	Δ fat mass %	Δ body mass (g)	Δ lean mass %	Δ fat mass %
WT	9.08 ± 1.83	−2.88 ± 3.87	4.19 ± 2.50	11.2 ± 4.46	−8.61 ± 4.32	8.81 ± 4.28
hAβ‐KI	11.7 ± 2.63^a^	−5.71 ± 2.54^a^	4.81 ± 2.53	12.3 ± 3.89	−8.25 ± 2.61	8.78 ± 2.54

^a^ *p* <0.05 versus WT. Comparisons were made within sexes using a Student's *t*‐test.

**FIGURE 1:**
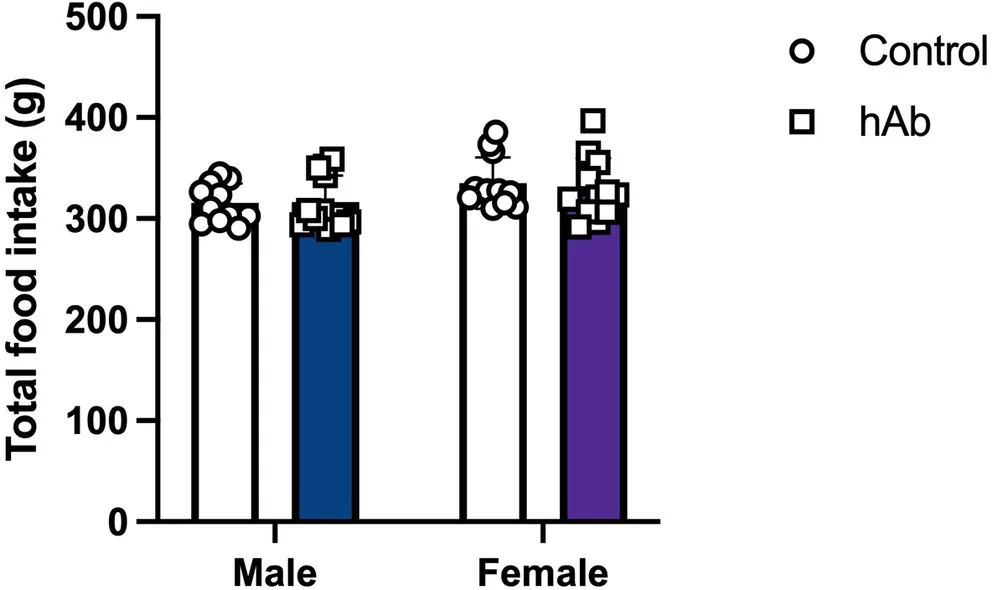
Total food intake. Food intake was measured weekly across the entirety of the study and summed to determine total food intake. All data presented as means ± SD.

### 3.2 EDL contractile function and fiber type across age and sex

We first explored the EDL, a primarily fast twitch muscle. In the 8‐month‐old male mice, there were no differences in EDL muscle mass, either absolute or relative to body mass or calculated CSA (Figure 2a–c). Using an isometric force‐frequency protocol to assess the isolated contractile properties of the EDL, we found no differences in force production between the 8‐month‐old WT and hAβ‐KI male mice, either in absolute or relative to muscle mass (Figure 2d,e). During the fatigue protocol, the 8‐month‐old male hAβ‐KI EDL showed a leftward shift in the percent of force produced over time curve, indicative of greater fatiguability compared to their WT counterparts, and supported by a significant time × genotype interaction; albeit the AUC and time to 60% rundown was not statistically significant (Figure 2f). A fiber type shift was not behind these differences in fatiguability as there were no differences in the percent of glycolytic and oxidative fibers, and no differences in fiber type specific CSA between the genotypes (Figure 2g–i). In the 15‐month‐old male mice, isolated EDL mass was not significantly different; however, when made relative to body mass, specific EDL mass was significantly smaller in the hAβ‐KI mice compared to WT mice, though there were no differences in isolated CSA (Figure 3a–c). Similar to the 8‐month‐old mice, there were no differences in force production between the 15‐month‐old WT and hAβ‐KI male mice during the isometric force‐frequency protocol. Contrary to the younger mice, there were no differences in the fatiguability of the EDL (Figure 3d–f). Additionally, there were no differences in the percent composition of glycolytic and oxidative muscle fibers, and no differences in fiber type specific CSA (Figure 3g–i). Together, these results suggest that the EDL of hAβ‐KI male mice fatigues faster than WT counterparts 8 months of age, and prior to cognitive deficit onset, but this difference is not present at 15 months of age.

**FIGURE 2:**
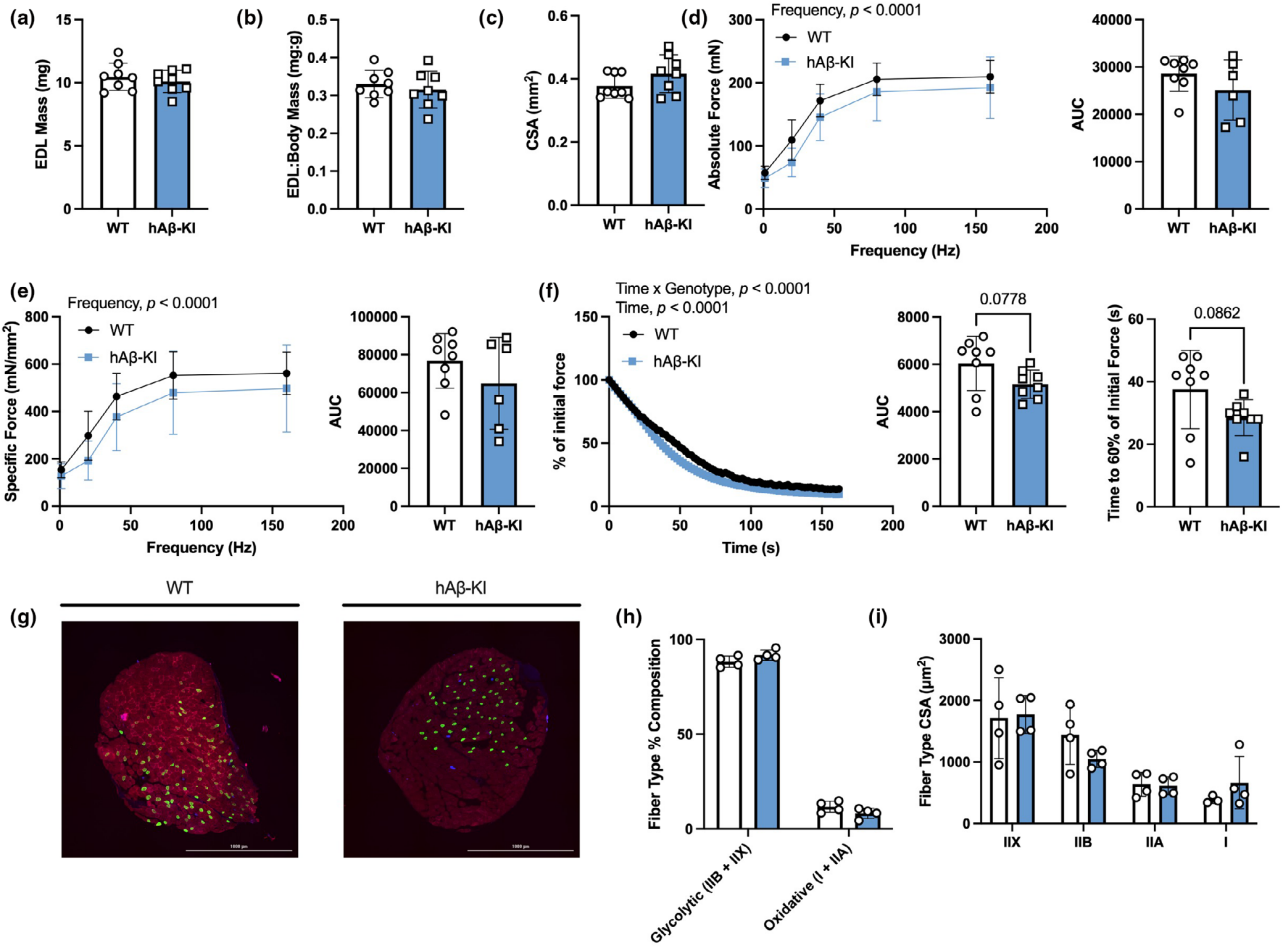
8‐month‐old male EDL characterization. (a) EDL mass in mg. (b) EDL mass (mg) relative to body mass (g). (c) Cross‐sectional area of the isolated EDL. (d) Absolute force production force frequency curve and corresponding AUC. (e) Specific force production force frequency curve and corresponding AUC. (f) Percent of initial force remaining throughout 3‐min isometric fatigue protocol and the corresponding AUC and time to reach 60% of the initial force in seconds. (g) Representative immunohistochemistry images of WT and hAβ‐KI EDL. (h) Percent fiber type composition. (i) Cross‐sectional area of individual fiber types.

**FIGURE 3:**
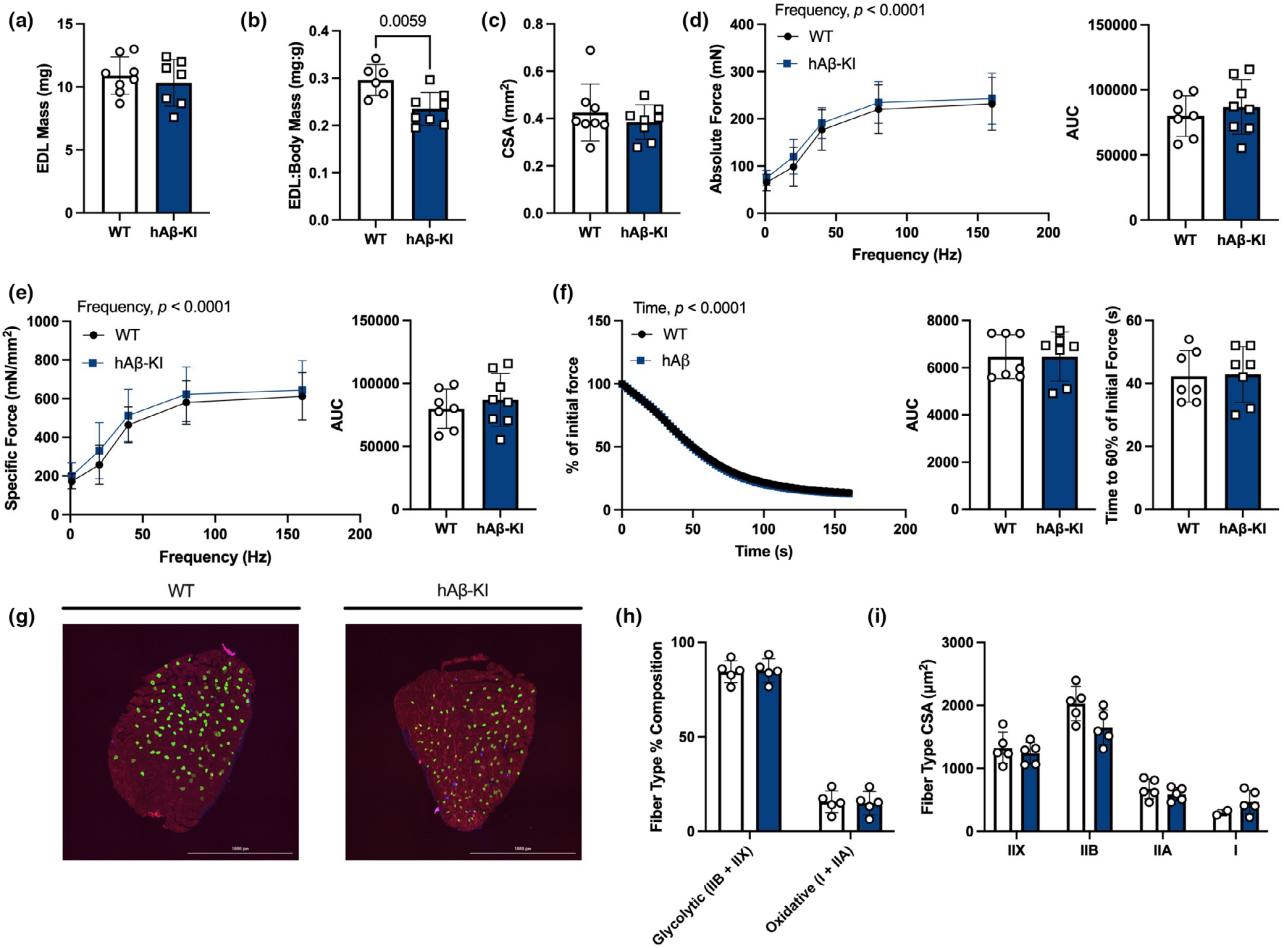
15‐month‐old male EDL characterization. (a) EDL mass in mg. (b) EDL mass (mg) relative to body mass (g). (c) Cross‐sectional area of the isolated EDL. (d) Absolute force production force frequency curve and corresponding AUC. (e) Specific force production force frequency curve and corresponding AUC. (f) Percent of initial force remaining throughout 3‐min isometric fatigue protocol and the corresponding AUC and time to reach 60% of the initial force in seconds. (g) Representative immunohistochemistry images of WT and hAβ‐KI EDL. (h) Percent fiber type composition. (i) Cross‐sectional area of individual fiber types.

In the 8‐month‐old female mice, isolated EDL mass was lower in the hAβ‐KI mice compared to WT mice, though this did not reach significance (*p* = 0.063; Figure 4a). Regardless, there was no significant difference in EDL mass relative to body mass, nor isolated CSA (Figure 4b,c). We found no differences between 8‐month‐old female hAβ‐KI and WT mice in either absolute or specific force production during the isolated‐force frequency protocol (Figure 4d,e). However, during the fatigue protocol, the hAβ‐KI mice exhibited a rightward shift in the percent of force produced over time curve, indicative of less fatiguability compared to their WT counterparts, which was supported by a significant time × genotype interaction (Figure 4f). This difference in fatiguability did not appear to be due to changes in fiber type as there were no differences in percent glycolytic or oxidative fibers, and there were no differences in fiber type specific CSA (Figure 4g–i). In the 15‐month‐old female mice, there were no differences in EDL muscle mass, in absolute, relative to body mass, or isolated CSA (Figure 5a–c). Similar to the 8‐month‐old mice, there were no differences in force production between the 15‐month‐old WT and hAβ‐KI female mice during the isometric force‐frequency protocol. Contrary to the younger mice, there were no differences in the fatiguability of the EDL (Figure 5d–f). Additionally, there were no differences in the percent composition of glycolytic and oxidative muscle fibers, and no differences in fiber type specific CSA (Figure 5g–i). Together, these results suggest that the EDL of hAβ‐KI female mice are protected against fatigue at 8 months of age; however, this appears to be lost at 15‐months of age.

**FIGURE 4:**
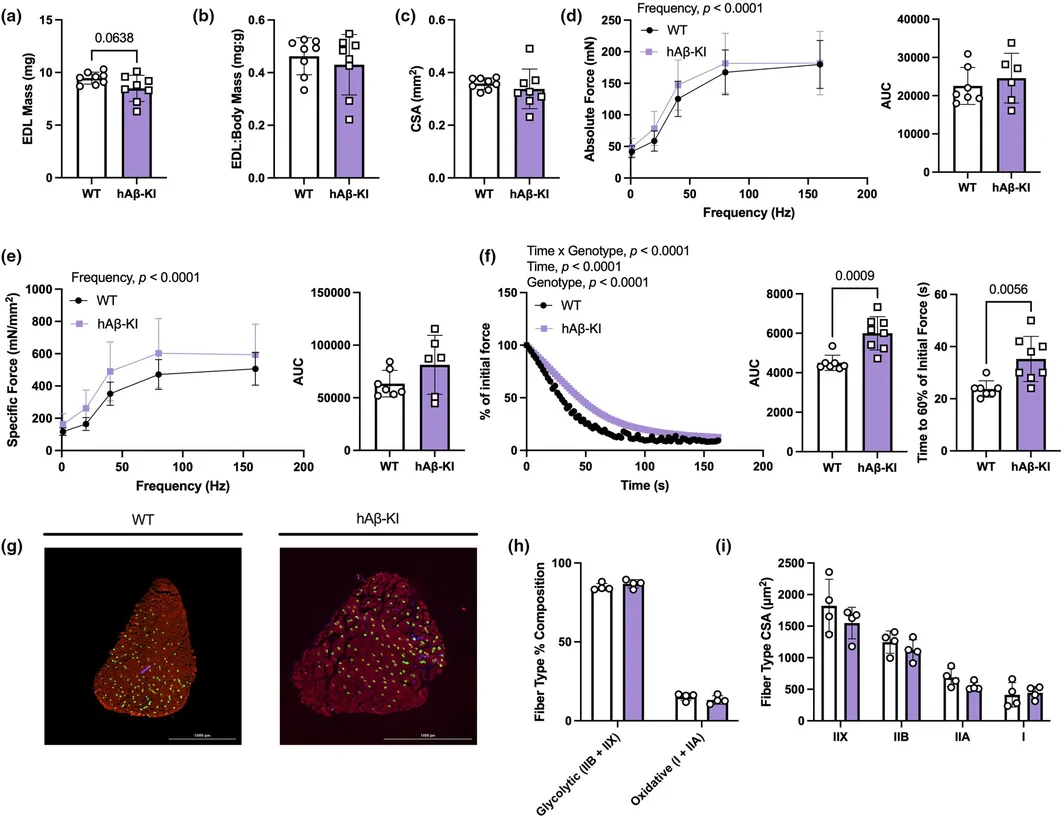
8‐month‐old female EDL characterization. (a) EDL mass in mg. (b) EDL mass (mg) relative to body mass (g). (c) Cross‐sectional area of the isolated EDL. (d) Absolute force production force frequency curve and corresponding AUC. (e) Specific force production force frequency curve and corresponding AUC. (f) Percent of initial force remaining throughout 3‐min isometric fatigue protocol and the corresponding AUC and time to reach 60% of the initial force in seconds. (g) Representative immunohistochemistry images of WT and hAβ‐KI EDL. (h) Percent fiber type composition. (i) Cross‐sectional area of individual fiber types.

**FIGURE 5:**
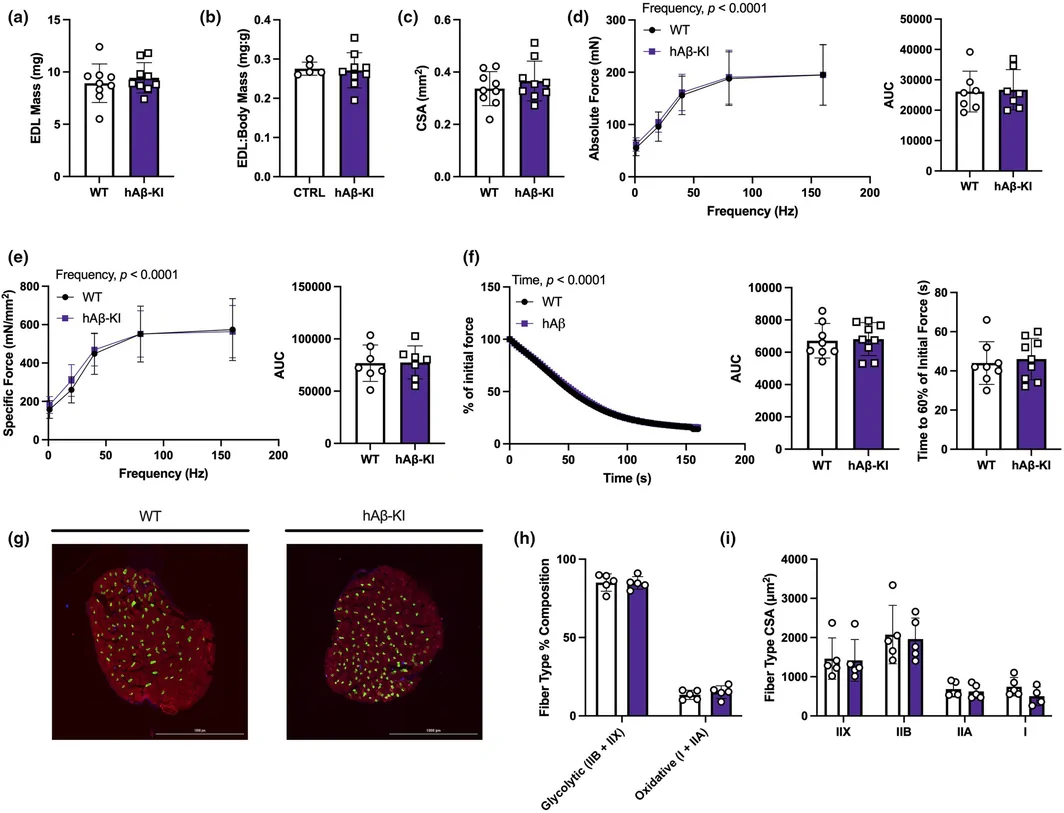
15‐month‐old female EDL characterization. (a) EDL mass in mg. (b) EDL mass (mg) relative to body mass (g). (c) Cross‐sectional area of the isolated EDL. (d) Absolute force production force frequency curve and corresponding AUC. (e) Specific force production force frequency curve and corresponding AUC. (f) Percent of initial force remaining throughout 3‐min isometric fatigue protocol and the corresponding AUC and time to reach 60% of the initial force in seconds. (g) Representative immunohistochemistry images of WT and hAβ‐KI EDL. (h) Percent fiber type composition. (i) Cross‐sectional area of individual fiber types.

### 3.3 Soleus contractile function and fiber type across age and sex

We next explored the soleus, a primarily slow‐twitch muscle. In the 8‐month‐old male mice, there were no differences in soleus muscle mass, either absolute, relative to body mass, or isolated CSA (Figure 6a–c). Additionally, there were no differences in force production between the 8‐month‐old WT and hAβ‐KI male mice during the isometric force‐frequency protocol, and there were no differences in the fatiguability of the soleus at 8 months of age (Figure 6d–f). Furthermore, there were no differences in the percent composition of glycolytic and oxidative muscle fibers, and no differences in fiber type specific CSA (Figure 6g–i). In the 15‐month‐old male mice, there were no differences in soleus muscle mass, either absolute, relative to body mass, or isolated CSA (Figure 7a–c). Similar to the 8‐month‐old mice, there were no differences in force production between the 15‐month‐old WT and hAβ‐KI female mice during the isometric force‐frequency protocol, and there were no differences in the fatiguability of the soleus (Figure 7d–f). Additionally, there were no differences in the percent composition of glycolytic and oxidative muscle fibers, and no differences in fiber type specific CSA (Figure 7g–i). Taken together, these results suggest that, unlike the EDL, the soleus is unaffected by the hAβ‐KI genotype in male mice.

**FIGURE 6:**
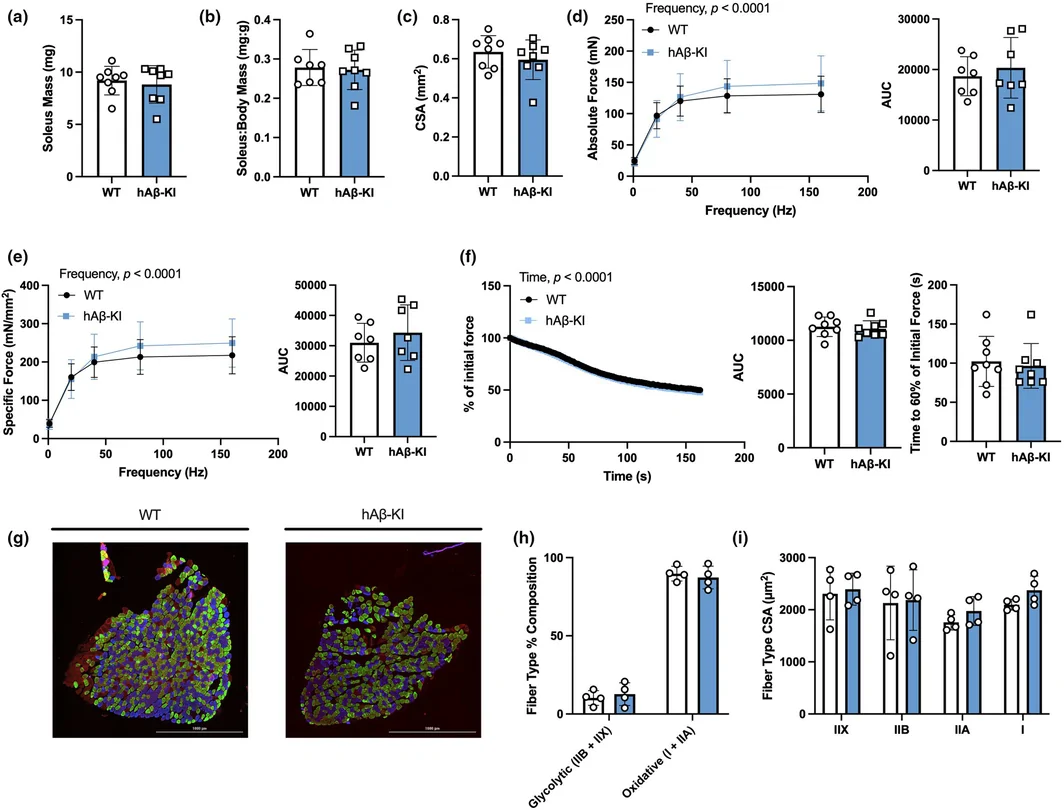
8‐month‐old male soleus characterization. (a) Soleus mass in mg. (b) Soleus mass (mg) relative to body mass (g). (c) Cross‐sectional area of the isolated soleus. (d) Absolute force production force frequency curve and corresponding AUC. (e) Specific force production force frequency curve and corresponding AUC. (f) Percent of initial force remaining throughout 3‐min isometric fatigue protocol and the corresponding AUC and time to reach 60% of the initial force in seconds. (g) Representative immunohistochemistry images of WT and hAβ‐KI soleus. (h) Percent fiber type composition. (i) Cross‐sectional area of individual fiber types.

**FIGURE 7:**
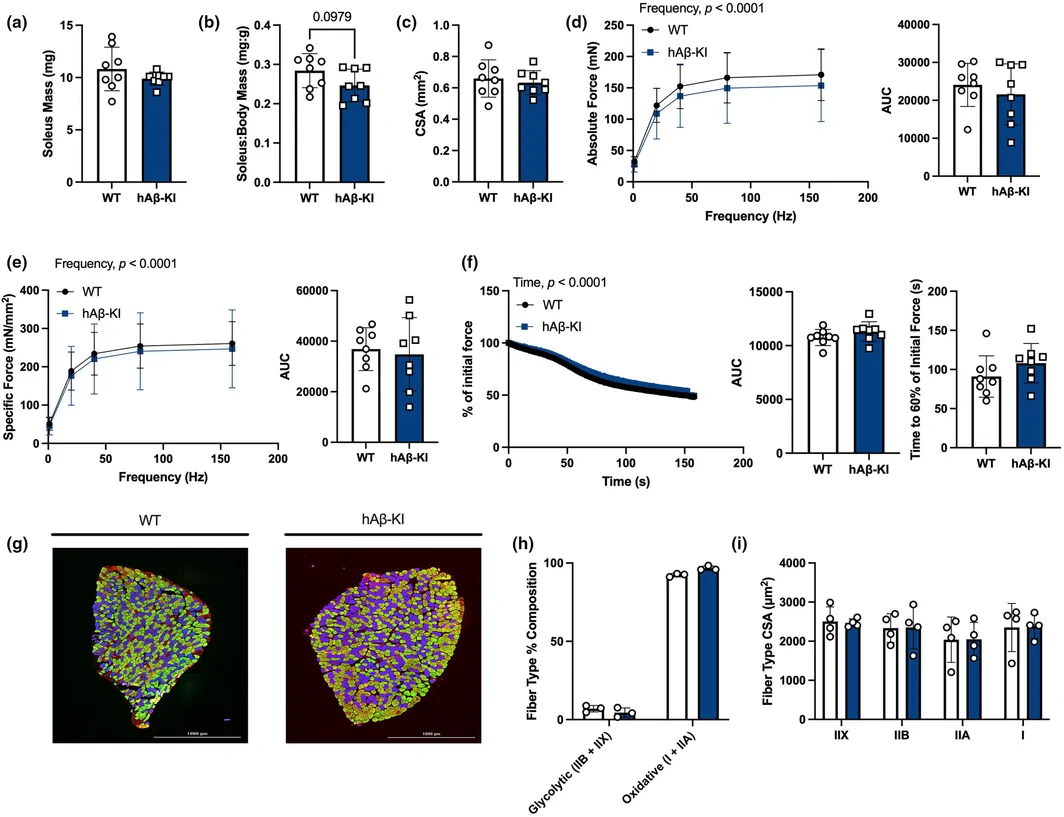
15‐month‐old male soleus characterization. (a) Soleus mass in mg. (b) Soleus mass (mg) relative to body mass (g). (c) Cross‐sectional area of the isolated soleus. (d) Absolute force production force frequency curve and corresponding AUC. (e) Specific force production force frequency curve and corresponding AUC. (f) Percent of initial force remaining throughout 3‐min isometric fatigue protocol and the corresponding AUC and time to reach 60% of the initial force in seconds. (g) Representative immunohistochemistry images of WT and hAβ‐KI soleus. (h) Percent fiber type composition. (i) Cross‐sectional area of individual fiber types.

In the 8‐month‐old female mice, there were no differences in soleus muscle mass, either absolute or relative to body mass, or isolated CSA (Figure 8a–c). During the isometric force‐frequency protocol, 8‐month‐old female hAβ‐KI mice produced significantly less force compared to their WT counterparts and resulted in a significant frequency × genotype interaction with post hoc testing in the two‐way repeated measure ANOVA showing differences between the hAβ‐KI and WT mice at 80 Hz and 160 Hz (Figure 8d). However, when made relative to soleus CSA, there were no longer any differences in specific force production between the female hAβ‐KI and WT mice at 8 months of age (Figure 8e). Similar to the EDL, during the fatigue protocol, the hAβ‐KI mice exhibited a rightward shift in the percent of force produced over time curve, indicative of less fatiguability compared to their WT counterparts, which was supported by a significant time × genotype interaction (Figure 8). Regardless of these functional alterations, there were no differences in percent composition of glycolytic and oxidative muscle fibers, and no differences in fiber type specific CSA (Figure 8g–i). In the 15‐month‐old female mice, there were no differences in soleus muscle mass, in either absolute, relative to body mass, or isolated CSA (Figure 9a–c). There were no differences in force production between the 15‐month‐old WT and hAβ‐KI female mice during the isometric force‐frequency protocol, and there were no differences in the fatiguability of the soleus (Figure 9d–f). Additionally, there were no differences in the percent composition of glycolytic and oxidative muscle fibers, and no differences in fiber type specific CSA (Figure 9g–i). Taken together, these results suggest that, though the 8‐month‐old female hAβ‐KI mice produce less force than their age‐matched WT counterparts, similar to the EDL, the soleus of hAβ‐KI female mice is protected against fatigue at 8 months of age; however, this appears to be lost at 15 months of age.

**FIGURE 8:**
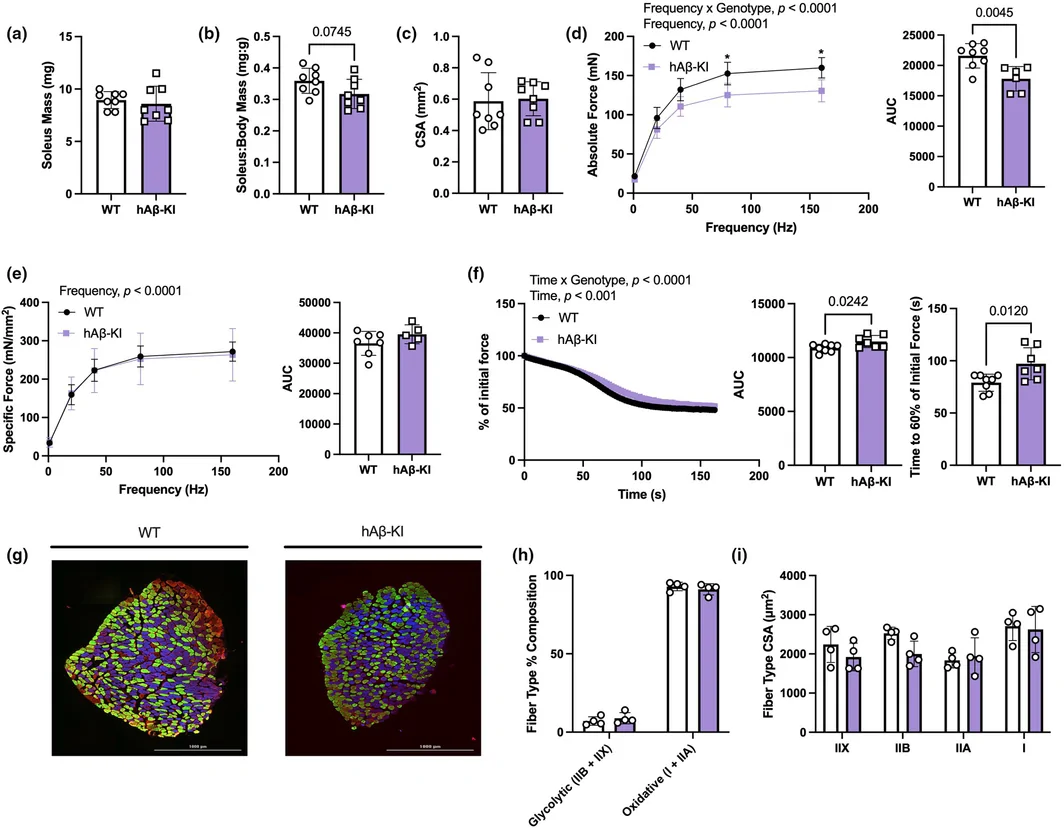
8‐month‐old female soleus characterization. (a) Soleus mass in mg. (b) Soleus mass (mg) relative to body mass (g). (c) Cross‐sectional area of the isolated soleus. (d) Absolute force production force frequency curve and corresponding AUC. (e) Specific force production force frequency curve and corresponding AUC. (f) Percent of initial force remaining throughout 3‐min isometric fatigue protocol and the corresponding AUC and time to reach 60% of the initial force in seconds. (g) Representative immunohistochemistry images of WT and hAβ‐KI soleus. (h) Percent fiber type composition. (i) Cross‐sectional area of individual fiber types.

**FIGURE 9:**
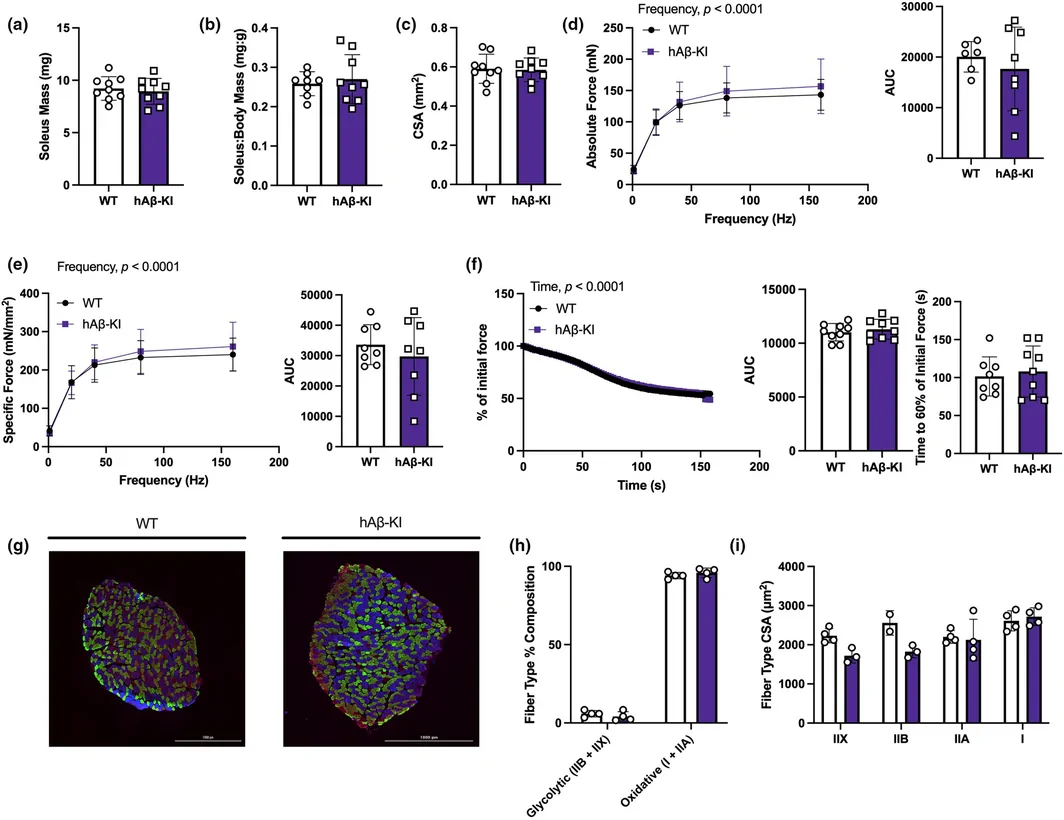
15‐month‐old female soleus characterization. (a) Soleus mass in mg. (b) Soleus mass (mg) relative to body mass (g). (c) Cross‐sectional area of the isolated soleus. (d) Absolute force production force frequency curve and corresponding AUC. (e) Specific force production force frequency curve and corresponding AUC. (f) Percent of initial force remaining throughout 3‐min isometric fatigue protocol and the corresponding AUC and time to reach 60% of the initial force in seconds. (g) Representative immunohistochemistry images of WT and hAβ‐KI soleus. (h) Percent fiber type composition. (i) Cross‐sectional area of individual fiber types.

### 3.4 Transcriptomic profile of EDL muscle at 8 months

To give some potential mechanistic insight to the observed differences in fatigue between the 8‐month‐old male and female hAβ‐KI and WT mice, RNA was extracted from the 8‐month‐old EDL for RNA‐Seq analysis performed by Novogene (Sacramento, USA). For the male mice, a total of 536 DEGs were identified in the male hAβ‐KI versus WT comparison which included 200 upregulated DEGs and 336 downregulated DEGs (Figure 10a). GO enrichment analysis of the DEGs between male hAβ‐KI and WT showed significant downregulation of pathways related to actin binding, actomyosin, myosin filament, musculoskeletal movement, muscle contraction, and muscle development (Figure 10b). Alternatively, a total of 437 DEGs were identified in the female hAβ‐KI versus WT comparison, with 218 being upregulated and 219 being downregulated (Figure 10c). GO enrichment analysis of the DEGs between female hAβ‐KI and WT mice did not show any downregulated pathways pertaining to muscle development or contraction and instead showed significant upregulation of pathways relating to aerobic respiration, proton motive force‐driven ATP synthesis, oxidative phosphorylation, and cellular respiration (Figure 10d). Corresponding well with these observed sex differences, only 86 DEGs overlapped between male and female comparisons (Figure 10e).

**FIGURE 10:**
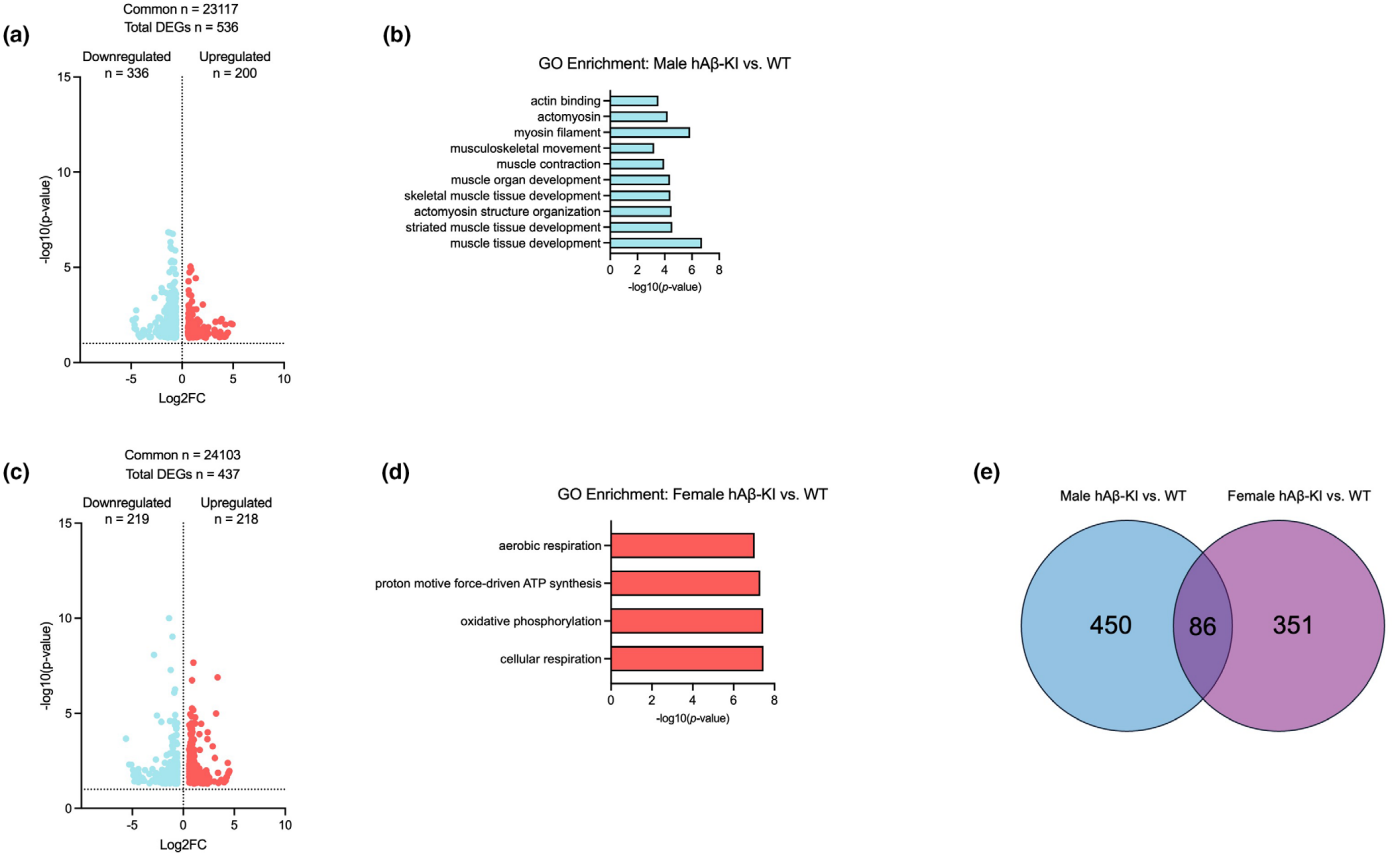
RNA Sequencing Summary in the EDL of 8‐month‐old male and female mice. (a) Volcano plots of differentially expressed genes identified from target comparisons between male WT versus male hAβ‐KI. The dashed line at y = 1.3 indicates the significance threshold (−log10(*p*) = 0.05; *p* < 0.05). (b) Selected top significant GO enrichment terms for male WT versus male hAβ‐KI. Exact *p* values for these genes can be found in Dataset S2. (c) Volcano plots of differentially expressed genes identified from target comparisons between female WT versus female hAβ‐KI. The dashed line at y = 1.3 indicates the significance threshold (−log10(*p*) = 0.05; *p* < 0.05). (d) Selected top significant GO enrichment terms for female WT versus female hAβ‐KI. Exact *p* values for these genes can be found in Dataset S4. (e) Venn diagram of DEGs identified in male and female comparisons.

## 4 DISCUSSION

This study characterized muscle form and function of 8‐ and 15‐month‐old male and female hAβ‐KI mice, a novel preclinical model of sporadic AD. Our findings demonstrate that male hAβ‐KI mice lost lean mass at a faster rate than WT controls, whereas no differences were observed in the females. Furthermore, isolated muscle analysis demonstrated that the 8‐month‐old male and female hAβ‐KI mice express altered fatiguability. Specifically, the EDL from 8‐month‐old male hAβ‐KI mice fatigued faster compared to their age‐matched WT counterparts, while both the EDL and the soleus of 8‐month‐old female hAβ‐KI mice fatigued slower compared to their WT controls. These sex differences were not caused by changes in fiber type but might result from variations in the transcriptomic response to the genetic modification of our model.

In elderly humans (aged 60 and older), accelerated loss of lean mass has been observed during the early stages of AD and associated with increased dementia risk and severity (Burns et al., 2010; Wu et al., 2025). Furthermore, sarcopenia, the age‐related loss of both muscle mass and function, has been noted to increase the risk of AD in humans (Beeri et al., 2021; Ogawa et al., 2018). Consistent with these clinical observations, male and female 5×FAD transgenic AD mice exhibit lower lean mass as early as 2 months of age compared to their WT counterparts (Marshall et al., 2024). Similarly, we found that male hAβ‐KI mice had a larger gain in body mass paired with a greater loss in lean mass percentage across the study, suggesting a potential sarcopenic phenotype; though in vivo muscle strength should be investigated in future studies.

To determine if muscle specific characteristics differ between genotype, we isolated and examined two differing muscle types, the EDL and soleus. The EDL is a primarily fast‐twitch muscle, offering greater force production, but fatigues quickly. Alternatively, the soleus is primarily a slow‐twitch muscle, offering greater fatigue resistance. We observed differing fatiguability of the isolated EDL muscle, in a sex specific manner. At 8 months of age, prior to the onset of cognitive deficits (Baglietto‐Vargas et al., 2021), the EDL of male hAβ‐KI mice fatigued faster compared to their controls, while the EDL of female hAβ‐KI mice fatigued slower. The EDL fatigue resistance of healthy male and female WT mice has been shown to be differentially affected by age, with males being more susceptible to fatigue at an earlier age than females (Hill et al., 2020). Given this, the hAβ‐KI males appear to be expressing an early‐onset aging phenotype compared to the females, who appear to be protected from such, especially as the soleus of the 8‐month‐old hAβ‐KI females fatigues slower than their WT controls. However, the exact mechanisms behind the protection of the female hAβ‐KI mice are yet to be uncovered. Furthermore, why the 8‐month‐old mice, but not the 15‐month‐old mice, exhibited functional alterations should be investigated further to determine if these early alterations are transient or whether age‐related declines in both genotypes mask any genotype specific effects at the later age.

To explore the potential mechanisms underlying these early functional differences, we performed RNA sequencing on skeletal muscle from the 8‐month‐old mice. Specifically, 8‐month‐old male hAβ‐KI mice exhibited significant downregulation of RNA related to skeletal muscle development and contraction compared to their WT counterparts, which alone are likely to contribute to the fatiguability of the EDL. Furthermore, actin binding is an essential component of muscle contraction and the sliding filament theory, in which the myosin head binds to actin, forming the crossbridge, to initiate the power stroke (Sandow, 1952). The interaction between actin and myosin has been suggested to be a major contributor to muscle fatigue (Cooke, 2007), and as such, may be a cause of the fatigue observed in the 8‐month‐old male hAβ‐KI EDL, though directed studies are required to explore this hypothesis. Alternatively, 8‐month‐old female hAβ‐KI mice showed no downregulation of skeletal muscle development and contraction; however, there was a significant upregulation of genes related to aerobic respiration and oxidative phosphorylation compared to their WT counterparts. Generally, slow‐twitch muscle fibers have a higher aerobic capacity and rely more heavily on aerobic respiration and oxidative phosphorylation than their fast‐twitch counterparts, making them more fatigue‐resistant (Allen et al., 2008; Wilson et al., 2012). Here, we found that the EDL from female hAβ‐KI mice exhibited upregulation of genes associated with aerobic respiration, potentially contributing to their enhanced fatigue resistance, even though our fiber‐type analysis, which depends solely on myosin heavy chain composition, showed no change, suggestive of underlying metabolic adaptations potentially paired with a fast‐to‐slow fiber type shift. Future studies should examine mitochondrial respiration across both sexes and genotypes, especially given the small sample size used for RNA sequencing analysis.

Sporadic AD is influenced by a multitude of interacting risk factors including diet (Moradi et al., 2020) and biological sex, specifically at the age of menopause onset (Mosconi et al., 2018), which may influence its onset and severity. It is therefore possible that the hAβ‐KI mouse requires additional environmental or physiological stressors to fully uncover skeletal muscle deficits comparable to those observed in humans and other transgenic models. In fact, a high fat diet has been shown to exacerbate both muscular and cognitive impairments in rodent models, including increased Aβ pathology and poorer muscle function (Lee et al., 2015; Mazzei et al., 2021). Similarly, sex hormones, such as estrogen, are known to influence both brain and muscle health, and their loss in females is associated with increased vulnerability to sarcopenia and cognitive decline (Hara et al., 2015; Pellegrino et al., 2022; Volpato et al., 2013). Given that female mice do not undergo menopause, this may partially explain the absence of a female‐specific phenotype in the present study. Future studies should therefore examine whether introducing external stressors, such as metabolic or hormone stressors, would unmask more pronounced muscle and cognitive phenotypes in the hAβ‐KI model.

This study provides several opportunities to further characterize the mechanisms underlying the observed differences in muscle function. However, there are important considerations when interpreting these findings in the context of human physiology. Firstly, rodents express a much higher percentage of fast‐twitch muscle fibers compared to humans, and as such, translation of these results to a human population should be made with caution. Secondly, this research primarily focused on fiber type expression as a potential explanation for adaptations to muscle fatiguability. However, previous research has shown denervated neuromuscular junctions in old male 3×TgAD mice and impairments to peripheral nerve activity in relatively young male and female (4 and 6 months old, respectively) 5×FAD mice, resulting in significant reductions in contractile and torque production (Brisendine et al., 2024; Xu et al., 2022). Therefore, future studies should examine peripheral nerve conductance and neuromuscular junction integrity in the hAβ‐KI mouse. Finally, while RNA sequencing provided an initial exploratory analysis of potential mechanisms behind the alterations to muscle fatigability in the hAβ‐KI mice, the sample size was relatively small and requires dedicated studies to conduct follow‐up analysis on pathways of interest.

Together, this preliminary characterization study suggests that the isolated EDL muscle of 8‐month‐old male and the EDL and soleus muscles of 8‐month‐old female hAβ‐KI mice exhibit altered fatigue resistance during isometric contraction, effects that were not present at 15 months of age. These alterations occurred prior to previously reported cognitive deficits in this model, highlighting a potential temporal relationship between muscle function and cognitive performance. Future studies should therefore investigate the relationship between muscle fatigue and cognitive performance in the hAβ‐KI mouse, as well as the underlying mechanisms driving these early differences in muscle function.

## AUTHOR CONTRIBUTIONS

**Emily N. Copeland:** Conceptualization; data curation; formal analysis; investigation; methodology; project administration. **Evelin Melekh:** Conceptualization; data curation; formal analysis; investigation; methodology. **Val Andrew Fajardo:** Conceptualization; formal analysis; funding acquisition; project administration; resources; supervision. **Rebecca E. K. MacPherson:** Conceptualization; formal analysis; funding acquisition; project administration; resources; supervision.

## FUNDING INFORMATION

This work was supported by Brock University Explore Grant and a Chancellor's Chair for Research Excellence Grant to R.E.K.M. and a Canada Research Chair Tier 2 award to V.A.F. E.N.C. was supported by a NSERC CGS‐D award. E.M. was supported by CIHR CGS‐M (2023–2024) and Ontario Graduate Scholarship awards (2024–2025).

## CONFLICT OF INTEREST STATEMENT

The authors declare no competing interests.

## ETHICS STATEMENT

All experimental procedures were approved by Brock University’s Animal Care Committee (AUP No. 23‐08‐02) and carried out in accordance with the Canadian Council on Animal Care guidelines.

## Supporting information


Dataset S1.

Dataset S2.

Dataset S3.

Dataset S4.

## Data Availability

Data supporting the findings of this work can be made available upon reasonable request to the corresponding author. The raw and processed RNA sequencing data used for DEG and GO analysis can be found at GEO #GSE343951.
